# Warming signals in temperate reef communities following more than a decade of ecological stability

**DOI:** 10.1098/rspb.2022.1649

**Published:** 2022-12-21

**Authors:** G. A. Soler, G. J. Edgar, N. S. Barrett, R. D. Stuart-Smith, E. Oh, A. Cooper, K. R. Ridgway, S. D. Ling

**Affiliations:** ^1^ Institute for Marine & Antarctic Studies, University of Tasmania, Private Bag 129, Hobart, Tasmania 7001, Australia; ^2^ CSIRO Hobart, Castray Esplanade, Battery Point Tasmania 7004, Australia

**Keywords:** climate change, functional traits, kelp, marine macroinvertebrates, reef fishes, tropicalization

## Abstract

Ecosystem structure and function are increasingly threatened by changing climate, with profound effects observed globally in recent decades. Based on standardized visual censuses of reef biodiversity, we describe 27 years of community-level change for fishes, mobile macroinvertebrates and macroalgae in the Tasmanian ocean-warming hotspot. Significant ecological change was observed across 94 reef sites (5–10 m depth range) spanning four coastal regions between three periods (1992–95, 2006–07, 2017–19), which occurred against a background of pronounced sea temperature rise (+0.80°C on average). Overall, fish biomass increased, macroinvertebrate species richness and abundance decreased and macroalgal cover decreased, particularly during the most recent decade. While reef communities were relatively stable and warming was slight between the 1990s and mid-2000s (+0.12°C mean temperature rise), increased abundances of warm affinity fishes and invertebrates accompanied warming during the most recent decade (+0.68°C rise). However, significant rises in the community temperature index (CTI) were only found for fishes, invertebrates and macroalgae in some regions. Coastal warming was associated with increased fish biomass of non-targeted species in fished zones but had little effect on reef communities within marine reserves. Higher abundances of larger fishes and lobsters inside reserves appeared to negate impacts of ‘thermophilization’.

## 1. Background

Globally, marine ecosystems are increasingly threatened by climate change, including ocean warming and alteration of ocean circulation [1]. Given that most marine creatures are ectothermic, global ocean warming is driving species redistributions *en masse* [2,3]. Marine species re-distributions include changes in local abundance of key functional species and cascading impacts across trophic levels [4,5]. Concerningly, climate change projections indicate accelerating warming. Thus, early signs of change will almost certainly continue and likely also accelerate [6,7].

Impacts of climate change on reef ecosystems vary regionally, and for temperate reefs, in particular, species composition is observed to change as warm water species migrate poleward and cool water species diminish or disappear [8–14]. Furthermore, increases in functional groups such as herbivores due to warming seas have been documented worldwide [4], with such trends readily observable from long-term underwater visual census (UVC) of shallow reef communities [15,16].

**Figure 1.**
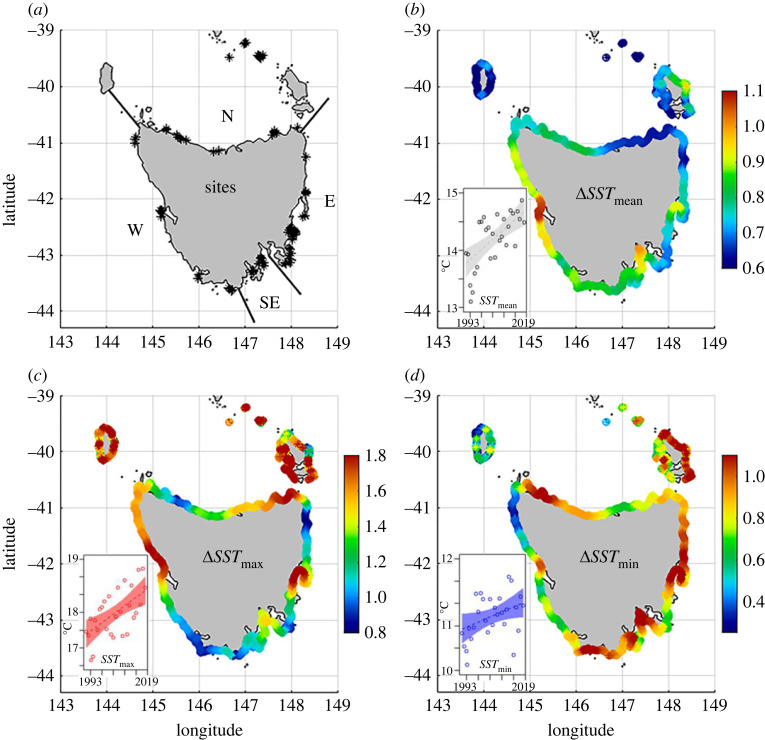
Map of Tasmanian study location and spatial variability in coastal warming. (*a*) Study sites (*n* = 94; asterisks denote sites) and regions (N: North; E: East; SE: Southeast; W: West), (*b–d*) spatial patterns of sea surface temperature (SST) warming (increase in ^o^C), by annual mean, maximum (max) and minimum (min), respectively, for 1000 points along the 5 m depth contour from 1993 to 2019. Inset plots show overall trends for Tasmania (for regional trends see electronic supplementary material, figure SM1). SST data were extracted from the Integrated Marine Observing System (IMOS) [25].

The temperate marine environment of south-eastern Australia, and Tasmania in particular, comprises an ocean warming hotspot [14,17,18]. Monitoring of sea temperature at the Maria Island Station in eastern Tasmania since 1944 has revealed a warming rate of 2.28°C/century, which is four times higher than the global average [17]. Tasmanian reefs are also among the most intensively surveyed by UVC methods worldwide, with prior studies spanning from 1990s to mid-2000s detecting an increasing presence of poleward range-shifting species [19,20]. Furthermore, cascading effects on marine communities, particularly in combination with non-climate stressors, have been documented along the Tasmanian coast [10,11,21,22]. While signs of pending collapse of reef communities were apparent in eastern Tasmania by the mid-2000s [21,22], coastal warming has accelerated across all regions of Tasmania [23,24] (figure 1; electronic supplementary material, figure SM1). During the most acute heatwave recorded in Tasmania from 2015 to 2016, above-average mortality of abalone (*Haliotis rubra*), die-back of bull kelp (*Durvillea potatorum*) and bleaching of crayweed (*Phyllospora comosa*) were observed [24].

Extensive Tasmanian studies comparing differences between no-fishing marine reserves and fished zones have revealed that fishing and climate change interactively affect ecological dynamics [14,26]. Cascading effects of ocean warming and overfishing of large predatory lobsters have been observed to cause phase-shifts from productive kelp beds to overgrazed and impoverished sea urchin barrens caused by a range-extending sea urchin [14,22,27]. Collectively, these studies have demonstrated that protected areas, with recovered predatory populations following cessation of fishing, tended to be more resilient to climate-driven stressors compared to fished reefs.

The complex interaction between protection, warming seas, wave exposure, regional differences and taxonomic communities can interrelate in different ways, with unique end results [14,19,28]. This study capitalizes on our unique dataset to explore the interactions of these factors in the patterns observed in the marine environment through time in Tasmania.

Here we analyse a multi-decadal SCUBA-based UVC reef community dataset to assess climate-driven changes in fish, invertebrate and macroalgal communities that we observed over 27 years of intense, yet variable, warming. Baseline UVC reef community data for all coastal regions of Tasmania were established in the early 1990s (1992–95) [29], then island-wide surveys were repeated in 2006–07 [19], and recently again in 2017–19 (herein reported). Specifically, we examined Tasmanian-wide and regional changes in the 27-year UVC dataset for each community type, and contrasted community changes during the 1990s to mid-2000s, with the mid-2000s to 2017–19. Also, we hypothesized that greater changes in reef communities would occur in the East, Southeast and North regions of Tasmania due to chronic warming caused by the well-documented southward extension of the East Australian Current (EAC) [17]. We also hypothesized that reefs protected from fishing would show greater resilience to climate-driven changes and that functional composition of reef communities would, as a symptom of ‘tropicalization’ (or more accurately referred to as thermophilization for higher-latitude temperate systems), shift toward increased herbivory, as observed for warmer-water reef ecosystems.

## 2. Material and methods

### (a) Data collection

A total of 94 reef sites (figure 1*a*; electronic supplementary material, table SM1) ranging from 5 to 10 m depth were consistently surveyed in each of three ∼decadal sampling periods, i.e. 1992–95, 2006–07 and 2017–19. Only one survey year per site in each period was included to achieve a balanced design through time. When multiple surveys were available in a survey period, the closest to the middle of the period was chosen. At each reef site, abundance and size structure (≥2.5 cm) of fishes, abundance of benthic macroinvertebrates (≥2.5 cm) and percent cover of macroalgae were concurrently censused along four contiguous 50 m transects laid end-on-end to span a total distance of 200 m along a fixed depth contour at either 5 m or 10 m depth (after Edgar and Barrett [30]). Sites were grouped into four geographical regions based on the divisions by Edgar *et al*. [29] and sea surface temperature (SST) regimes (figure 1*b–d*), which indicate unique environmental conditions and distinct biogeographical patterns in marine communities.

#### (i) Fishes

Abundances and size classes of fishes within 5 m at each side of the four 50 m transect lines were recorded *in situ* by divers (total area of 2000 m^2^; see Edgar & Barrett [30] for methodological details). The four transect lines were laid one after the other in a continuous line following the same depth contour. Schooling pelagic species (atherinids, clupeoids, arripids, mugilids, carangids) were removed for analyses due to their highly patchy and transient nature in the study region, with study focus on trends in reef-associated species. The biomass of excluded pelagic species comprised 4.6% of total fish biomass. Moreover, no significant increase in the biomass of pelagic fishes through time was evident around Tasmania through the study period.

In order to convert abundance data into a useful community measure [30], fish length observations were converted to biomass using species-specific length–weight relationships provided by *FishBase* [31]. The fish size bins increased with length, starting with 2.5 cm increments to 15 cm, then increasing in 5 cm increments to 50 cm and then 12.5 cm increments [30].

#### (ii) Macroinvertebrates

Abundances of macroinvertebrate species were scored within a 1 m lane along the four 50 m transects at each site (after Edgar and Barrett [30]). Biomass was not calculated for macroinvertebrates since size estimates were not recorded for all species during all survey periods.

#### (iii) Macroalgae

Focusing on multi-decadal change in large brown ‘habitat-forming’ laminarian and fucoid macroalgae, percent cover of macroalgal species was quantified by divers placing a total of five 0.25 m^2^ quadrats at 10 m intervals along each of the four 50 m transects at each site (after Edgar & Barrett [30]). Algae intersecting 50 equidistant points on each quadrat were counted in layers, with percent cover of overstory species recorded first, then pushed aside to expose the understory species for scoring. Thus, total macroalgae can exceed 100% cover within a quadrat.

#### (iv) Functional traits

Functional traits for fishes and macroinvertebrates were allocated to each species from previously published datasets [32] (electronic supplementary material, tables SM2, SM3 and SM4) and used as vector overlays for multivariate analyses. The functional traits for fish and macroinvertebrates were based on a trophic categorization, namely: higher carnivores (only for fishes), invertivores, omnivores, herbivores and planktivores. For macroalgae the functional traits were palatability (low, medium, high assigned from *in situ* observations of consumption by fishes and sea urchins), maximum height, maximum length and degree of branching (flat branching, radial branching, highly complex radial branching of primary, secondary branches and branchlets). Height and length traits were rounded to the nearest 0.05 m, 0.25 m and 1.00 m for understory, mid-canopy and overstory species, respectively. Functional trait categories were assigned by authors of this paper with algal expertise and extensive experience conducting field experiments examining herbivory in Tasmania (SDL, GJE, EO).

#### (v) Thermal mid-point (MP) of reef species

The thermal mid-point (MP) of species' realized thermal distributions was used as a measure of their thermal affinity [33]—a measure akin to the species temperature index. We used previously published values of thermal MPs for fishes and invertebrates, which were based on an extensive dataset of sea temperatures across their global distributions [34,35] (electronic supplementary material, table SM2 and SM3). The thermal MP of each algal species was estimated based on its published distribution [36–39] and by extracting the mean SST from Bio-ORACLE [40] using the 2002–2009 running mean for each set of coordinates, then estimating the mid-point.

#### (vi) Community temperature index

The community temperature index (CTI) was used to assess community-level responses to multi-decadal variations in temperature [33,34,41]. We calculated the CTI for the fish, invertebrate and macroalgal communities separately based on the mean of species-specific thermal MP weighted by their abundances (natural logged for fish and invertebrates) and percentage cover (for algae, untransformed) (see electronic supplementary material for derivation of the CTI equation).

#### (vii) Sea surface temperature data

SST data were sourced from a high-resolution product provided by the Integrated Marine Observing System (IMOS) [25]. For more information on data extraction, see electronic supplementary material: Sea Surface Temperature Data.

#### (viii) Statistical analyses

*Shifts in taxonomic structure of reef communities.* Analysis of temporal shifts in community structure was based on the 94 sites consistently surveyed during the three periods (electronic supplementary material, table SM1). For all analyses, the data from the four transects were pooled at the site level. To investigate for any ‘*Protection*’ effect, a subset of dedicated long-term reference sites in fished zones (*n* = 23) were designated as control sites, matched with nearby marine reserves sites (no-take zones) (*n* = 23) by depth, substratum and exposure. The control sites were in most cases between 1 km and 40 km from the matched marine reserve sites (electronic supplementary material, table SM1, Figure SM1b). These 46 sites constituted a balanced design for testing for the effect of *Protection.* For this subset of sites, we tested whether *Protection* interacted significantly with *Period* or *Region* for Tasmanian reefs for any reef community type before running any further statistical tests.

Shifts in taxonomic structure for each community type were examined using non-metric multidimensional scaling (nMDS) and PERMANOVA using PRIMER6+. Similarity matrices were based on Bray–Curtis dissimilarity of the transformed Ln(*x* + 1) (cover for algae untransformed) data and PERMANOVAs used 9999 permutations. Regional centroids based on site means are presented as plots.

*Shifts in biodiversity metrics and community iemperature index (CTI).* We examined the effect of ‘Period’ (3 levels: 1992–95; 2006–07; 2017–19) and ‘Region’ (4 levels: North; East; Southeast; West) on shifts in biodiversity metrics (i.e. species richness, abundance and biomass for fish and macroinvertebrates, and % cover for macroalgae) plus CTI at the site-level for each reef community type using fixed effects two-way ANOVAs. *A priori* comparisons of the Period effect were conducted within each Region and statistical groupings identified using Tukey's ‘honest significant difference’ (HSD) method. We first used box plots and then ANOVAs to respectively visualize more clearly where the changes were occurring and if these changes were statistically significant.

Following 2-way ANOVAs, we then examined the effects of change (first ‘1992–'95’ to last ‘2017–'19’ periods) in SST (mean, max and min) and wave exposure on the changes in reef community metrics over the same time period, including change in relative abundance of species by thermal MP using linear models (for the full model, see electronic supplementary material: Statistical Analyses). For the linear models, we estimated the confidence intervals and effect of each variable on the univariate response variables. Wave exposure was categorized on a scale from 1 to 10 for each site, with 1 = sheltered to 10 = fully exposed to large ocean swell based on site observations by the authors. All univariate statistical tests and plotting were performed in *R* [42].

We examined the effect of ‘*Protection’* from fishing and the potential interactions between *SST* metrics and *wave exposure.* To this end, we ran analyses with the subset of reserved (*n* = 23: no-take areas) and control (*n* = 23) sites in fished zones. Interactive effects between the predictors of interest (i.e. Δ*SST*_mean_, Δ*SST*_min_, Δ*SST*_max_ and *Exposure*) and ‘*Protection*’ were checked; if there were nil interaction effects (*p*-value > 0.05), all protected and unprotected sites were pooled. In the case of significant interaction effects, fished and protected zones were modelled separately, while using the same linear model structure as described in electronic supplementary materials: Statistical Analysis.

*Changes in relative abundance of species by thermal mid-point (MP).* To explore the influence of SST on multi-decadal change in fish, invertebrate and macroalgae, we examined the relation of the thermal MP of a given species against the relative change in abundance (fish and macroinvertebrates) or percent cover (macroalgae). Relative change in abundance was estimated as the mean abundance (or percent cover for macroalgae) of each species across sites in 2017–'19 minus their mean abundance (or percent cover) in the early 1990s divided by the sum for the two periods × 100. Relative change in abundance for each species was then regressed against the thermal MP of each species using linear models (package *lme4* from *R* [43]) and the slope compared to zero (i.e. no change). This analysis was only performed for species recorded on at least 5% of sites in each region to avoid biases caused by rare species, which were stochastically observed in any given period due to low probability of encounter, and for which any change metrics may be unreliable. This method removed 66 of 115 species of fish (49 retained), 49 of 84 species of macroinvertebrates (35 retained) and 6 of 37 species of macroalgae (31 retained).

To assess if there was an effect of protection on multi-decadal changes in relative abundance of fish and macroinvertebrates, or percent cover of macroalgae, we ran linear models with the interaction effect of ‘*Protection’*. Since the interaction effect was not significant in any of the cases, data were pooled across '*Protection*’ status for subsequent analysis.

## 3. Results

### (a) Shifts in taxonomic structure of reef communities

Community structure of reefs did not interact with *Protection* in the subset of sites examined (electronic supplementary material, tables SM6, SM7 and SM8). Therefore, community analyses were based on the full dataset of 94 sites for each of the fish, macroinvertebrate and macroalgal communities.

**Figure 2.**
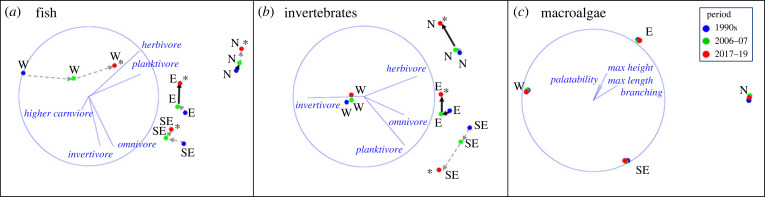
Multi-decadal shifts in reef community structure in nMDS space for (*a*) fishes, (*b*) mobile macroinvertebrates and (*c*) macroalgae. Asterisks denote significant multi-decadal shifts in community structure, bold arrows denote significant decadal shifts between particular sampling periods (*p* < 0.05). Symbols denote regional centroids. Correlations between community shifts and functional traits are overlaid as blue vectors. N: North; E: East; SE: Southeast and W: West. Max height and max length (m): maximum height or length of macroalgae, in metres.

Significant multi-decadal changes were observed for Tasmanian reef fishes and invertebrates, with significant differences in magnitude of change occurring between regions (figure 2; electronic supplementary material, table SM5). Variation between the periods 2005–'06 and 2017–'19 was generally greater than between the 1990s and 2005–'06 periods, with the notable exception of fishes in the West, which showed major change between each of the three periods, with increasing similarity to fish communities in other regions.

For fishes, significant multi-decadal shifts occurred in each of the four regions, with greatest jumps between all periods in the West, and between the second and last period in the East (figure 2*a*; electronic supplementary material, table SM5). Overlaid vectors of functional traits showed increasing importance of herbivores and planktivores to fish community structure in the latest period (figure 2*a*). Focussed analyses of biomass change in each trophic group also confirmed a significant state-wide increase in herbivorous fish biomass in the last period, and an increase in planktivorous fish biomass in the West (electronic supplementary material, figure SM2a,b; refer to species-specific trends in electronic supplementary material, table SM2).

Significant multi-decadal shifts also occurred in macroinvertebrate communities in three out of the four regions, the exception being the West, where fewer mobile invertebrates occur and negligible change was observed (figure 2*b*; electronic supplementary material, table SM5). Overlaid vectors of macroinvertebrate trophic groups indicate slight shifts toward a greater representation of herbivores in the latest period in the North and East, and more planktivores and invertivores in the Southeast (figure 2*b*). Macroinvertebrate herbivore abundance declined significantly state-wide, although grazing sea urchins (*Heliocidaris erythrogramma* and *Centrostephanus rodgersii*) remained relatively constant (electronic supplementary material, figure SM2f). Invertivorous macroinvertebrates decreased state-wide, while planktivorous (filter-feeding) macroinvertebrates declined in the North during the most recent period (electronic supplementary material, figure SM2d,e; for species-specific trends of fish and invertebrates see electronic supplementary material, table SM3).

Macroalgal communities remained relatively stable through time and did not show any clear shifts in functional traits (figure 2*c*; electronic supplementary material, table SM5; for macroalgal species-specific trends see electronic supplementary material, table SM4).

### (b) Shifts in species richness

**Figure 3.**
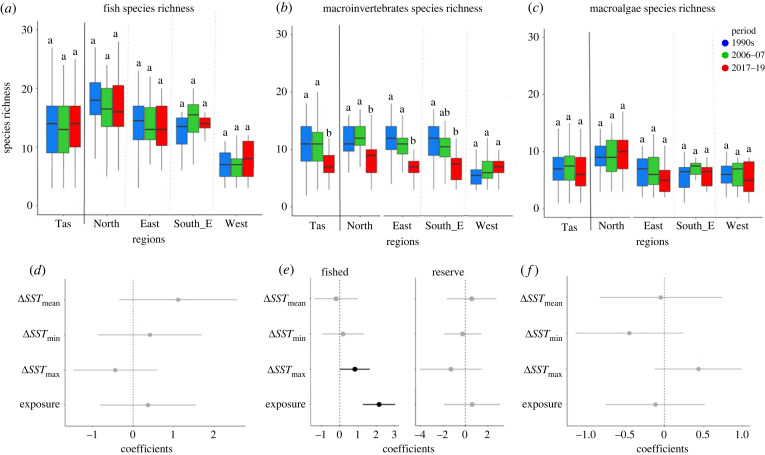
Multi-decadal trends in species richness for (*a*) fish, (*b*) macroinvertebrates and (c) macroalgae in each region of Tasmania. For boxplots, midline is the median, while upper and lower limits of the box depict the third and first quartiles (75^th^ and 25^th^ percentiles), respectively, and whiskers extend 1.5× times the interquartile range from the box to the furthest datum within that distance. Shared letters above boxplots indicate non-significant (*p* > 0.05) differences. Values are expressed per 2000 m^2^ for fish, 200 m^2^ for macroinvertebrates and 5 m^2^ for macroalgae. Lower plots (*d–f*) show coefficients derived from linear models examining multi-decadal (last minus first period) change in species richness for fish, invertebrates and macroalgae, respectively. Fixed effects (i.e. Δ*SST*_mean_, Δ*SST*_min_, Δ*SST*_max_, which all increased) are also based on multi-decadal change. In the case of significant interaction effects between protection and the environmental covariates, fished and reserved zones were modelled separately. Fixed effects were scaled to allow direct comparison of coefficients. Grey circles and bars indicate an effect overlapping zero (95% confidence). Black circles and bars indicated significant effects.

Fish species richness was relatively stable through time and did not appear to be significantly affected by warming (figure 3; electronic supplementary material, table SM9 and figure SM3). Species richness of macroinvertebrates significantly declined during the most recent decade (−27% state-wide between 1990s and 2017–19) except in the West, where a (non-significant) 20% increase in species richness occurred between 1990s and 2017–19 (figure 3*b*). Significant effects of protection on species richness of macroinvertebrates were revealed in some cases when using the subset of sites to determine any interaction effects between protection and environmental predictors. Therefore, linear models were run separately for the fished zones and reserves. For the fished zones, change in *SST*_max_ and *wave exposure* were positively associated with increase in species richness (figure 3*e*). Examination of Draftman's plots (electronic supplementary material, figures SM5 and SM6) revealed reefs with smallest increase in Δ*SST*_max_ in fished zones lost more species (negative change in species richness). Furthermore, sheltered reefs in fished zones also lost more species than wave exposed reefs. This can in part be attributed to the fact that more sheltered reefs had more species of macroinvertebrates to begin with, so relative loss here was greatest. None of the covariates used in the linear model had significant effects on protected reefs for macroinvertebrates species richness (figure 3*e*; electronic supplementary material, table SM9).

Macroalgal species richness across Tasmania was relatively stable through time (figure 3; electronic supplementary material, table SM9).

### (c) Shifts in fish biomass, invertebrate abundance and macroalgal cover

**Figure 4.**
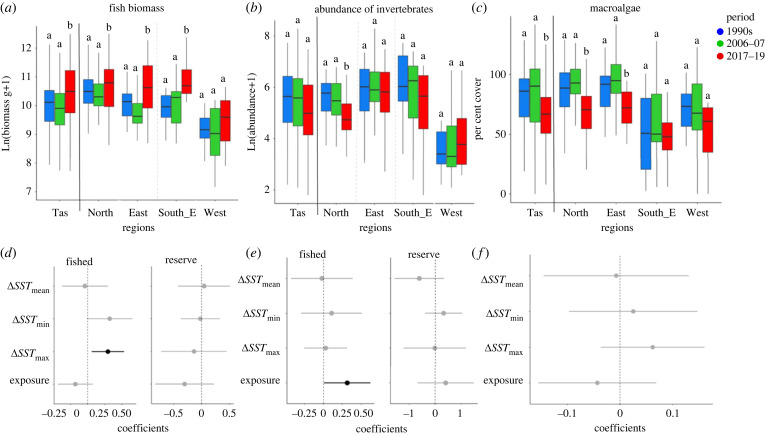
Multi-decadal trends in (*a*) fish biomass, (*b*) macroinvertebrate abundance and (*c*) macroalgal percent cover in each region of Tasmania. Lower plots (*d–f*) show coefficients derived from linear models examining multi-decadal (last minus first period) change in biomass and abundance, respectively; plot features and linear modelling as described in the figure 3 caption.

Fish biomass significantly increased (+50% between 1990s and 2017–19) across Tasmania during the most recent period (N: +5%, E: +86% and SE: +166%; between 1990s and 2017–19), the exception being the West (figure 4*a*). Interactive effects between *Protection* and environmental predictors were significant in some cases, thus linear models were run separately for marine reserves and fished zones. For fished zones, warming *SST*_max_ was positively related to fish biomass (figure 4*d*, electronic supplementary material, figure SM3 and table SM10).

Invertebrate abundance decreased across Tasmania by −31% (and −47% in the South-east), although only the decline in the Northern region (−54% between 1990s and 2017–19; figure 4*b*) was statistically significant. Significant interactions were detected between *Protection* and some covariates, so linear modelling was conducted separately for fished and protected (reserve) reefs (figure 4*e*; electronic supplementary material, figures SM4 and SM6; table SM10). Abundance of invertebrates in fished zones decreased more at sheltered sites than in wave-exposed sites.

Macroalgal cover decreased significantly (−14% state-wide between 1990s and 2017–19, and −17% in N, −15% in E; figure 4*c*). However, none of the covariates in the linear models adequately explained this decline (figure 4*f*; electronic supplementary material, table SM10).

### (d) Shifts in community temperature index

**Figure 5.**
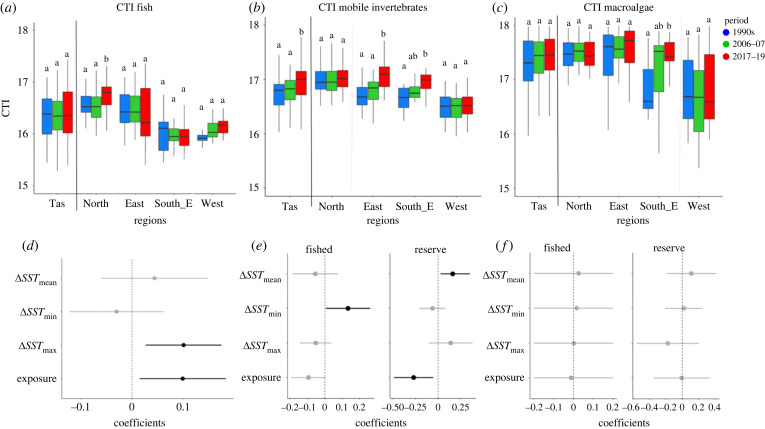
Multi-decadal patterns in community temperatureindex for (*a*) fish, (*b*) invertebrate and (*c*) macroalgal communities for Tasmania and each region. CTI for the fish, invertebrate and macroalgal communities was calculated separately based on the mean of species-specific thermal MP weighted by their abundances. Lower plots (*d–f*) show coefficients derived from linear models examining multi-decadal (last minus first period) change; plot features and linear modelling as described in the figure 3 caption.

CTI for fishes did not change significantly through time across Tasmania, but regional warming of the community in the North (figure 5*a*) was significant and positively related to *SST*_max_ (figure 5*d*; electronic supplementary material, table SM11). CTI for fishes also increased at wave exposed sites (figure 5*d*). Macroinvertebrate CTI showed significant warming (+0.23°C state-wide between 1990s and 2017–19, +0.38°C in the E, +0.37°C in the SE; figure 5*b*). Although the effects of covariates depended on ‘*Protection*’; for fished reefs, warming macroinvertebrate CTI was related to warming *SST*_min_ (figure 5*e*; electronic supplementary material, table SM11), while warming macroinvertebrate CTI on protected reefs was significantly related to warming *SST*_mean_ (figure 5*e*; electronic supplementary material, table SM11). Change in CTI for macroinvertebrates on reefs protected from fishing was negatively related with wave exposure, meaning more sheltered sites had greater increases in CTI. Large brown macroalgal CTI remained relatively stable at the Tasmanian level, however there was significant warming of macroalgal CTI in the Southeast region between the first and last period (figure 5*c,f*, electronic supplementary material, table SM11). No significant correlations were found between changes in macroalgal CTI and any of the covariates examined.

### (e) Changes in relative abundance of species by thermal MP

**Figure 6.**
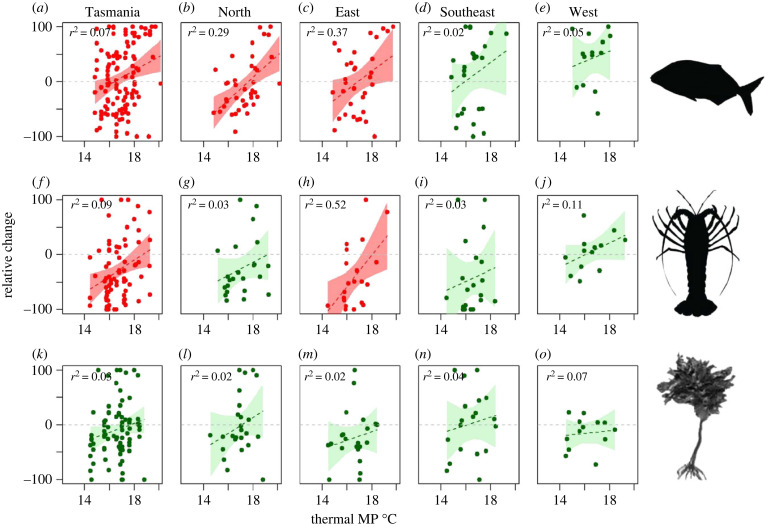
Multi-decadal change in relative abundance of fishes (*a–e*), macroinvertebrates (*f–j*) and percent cover of macroalgae (*k*–*o*) against their thermal MP. Relative change was estimated as the mean abundance or percent cover for each species across sites in the most recent sampling period (i.e. 2017–19) minus their mean abundance or percent cover in the first decade (i.e. 1992–95), expressed as a percentage. Thermal MP °C is the mid-point of the 5^th^–95^th^ percentile of species' thermal MP. Data were fitted with linear models; 95% confidence intervals are shown by green and pink shading. Pink shading indicates significant relationships (*p* < 0.05), green shading indicates non significance. *R*-squared correlations are shown for each plot in the top left corner. Values expressed as change per 2000 m^2^ for fish, 200 m^2^ for macroinvertebrates and 5 m^2^ for macroalgae.

Despite relatively few significant changes in thermal MPs at the community level using CTI, multi-decadal change in relative abundance of individual species was positively related to increasing thermal MP of fishes, macroinvertebrates and macroalgae across Tasmania (figure 6*a–o*). For fishes, regression of relative change in abundance against thermal MPs showed significant positive relationships (figure 6*a–c*). Although the proportions of fish species that increased or decreased in abundance were similar in some regions (indicated by the similar numbers of fish species above and below the zero line; figure 6*b,c*), decreases in cooler-affinity species were balanced out by increases in warmer-affinity species. In the Southeast and West regions, however, increases in abundance dominated and did not depend on the thermal MPs of the species (figure 6*d,e*; see electronic supplementary material, table SM12 for full list).

Changes in macroinvertebrate abundances were also positively related to their thermal MPs, overall and for the Eastern region (figure 6*f,h*). A higher proportion of macroinvertebrate species decreased in abundance than increased, except for in the West (figure 6*g–j*). The few species that increased in relative abundance across all regions tended to have warmer thermal MPs (figure 6*f–j*; electronic supplementary material, table SM13).

Change in percentage cover of macroalgal species was positively related to thermal MPs overall, but this was not significant despite positive trends (figure 6*k–o*; electronic supplementary material, table SM14). More species of macroalgae decreased in percent cover in the East and West, indicated by a greater number of species below zero (figure 6*m,o*).

A sensitivity analysis revealed little change in linear model results when rare species (present at between 5% and 10% of sites) were excluded, albeit statistical power and associated probability values generally declined in model output. For fish, exclusion of rare species did not affect the trend or statistical significance between the change in relative abundance and thermal MPs (*p* < 0.005, *n* = 103 for 0% cut-off; *p* = 0.004, *n* = 47 for 5% cut-off; *p* = 0.04, *n* = 39 for 10% cut-off). For invertebrates, trends between the change in relative abundance and species thermal MPs for the whole of Tasmania crossed the *p* = 0.05 significance threshold when using the 5% cut-off (*p* = 0.06, *n* = 70 for 0% cut-off; *p* = 0.009, *n* = 34 for 5% cut-off; *p* = 0.19, *n* = 23 for 10% cut-off). By contrast, macroalgal trends showed significant increase Tasmania-wide when no species were excluded from the linear model (*p* = 0.003, *n* = 37 for 0% cut-off; *p* = 0.1, *n* = 31 for 5% cut-off; *p* = 0.06, *n* = 25 for 10% cut-off). Inclusion of rare species thus tended to increase power while also adding statistical noise to the analysis because of greater stochastic variability in relative abundance among rare species between time periods relative to common species, but with little change in direction and slope for each of the three communities for Tasmania overall.

## 4. Discussion

### (a) Shifts in structure of reef communities

Our visual censuses of Tasmanian sub-tidal reef communities over 27 years spanned a period of substantial coastal warming, with some reefs warming by >1°C from the early 1990s to recent years (figure 1). By global standards, the pace of marine climate change in Tasmania is extremely rapid. It was associated with long-term directional shifts in reef community structure, with the exception of large brown macroalgae. While analysis of community structure revealed clear shifts, these were not consistent across taxa or through space and time. Most pronounced were the changes in reef communities during the most recent decade following relative stability in the preceding decade, as reported by Stuart-Smith *et al*. [19]. Despite some warming in reef communities identified across Tasmania in the initial study, community structure was generally stable, an outcome attributed to a combination of: (1) the two snapshot surveys missing influences of temperature peaks in the intervening years and consequently not capturing the more dynamic species-level changes that followed the cyclical warming trend; and (2) relatively little overall warming occurring between the 1990s and mid-2000s (see electronic supplementary material, figure SM1), hence species-level trends had not yet translated to major change in community structures. The changes observed over the second decadal period (mid-2000s to 2017–19), concomitant with accelerated warming, suggest that some of these changes can occur when critical thermal thresholds are surpassed [10,21,44], or when the accumulation of multiple species-level changes reduces overall community resilience.

Confirming expectations of increased climate-driven impacts in the North, East and Southeast regions due to increasing influence of the EAC [17], these regions experienced pronounced coastal warming and distinct changes in fish and invertebrates communities. Despite the West coast also experiencing considerable warming, the changes in the reef communities here were less pronounced (except for fish in the nMDS analysis), possibly due to reduced larval supply from northern warmer-water reefs via the west coast Zeehan Current relative to the EAC capable of transporting greater volume of water and organisms. On the east coast in particular, we confirmed expectations of tropicalization, in that observed changes in community functional traits shifted toward increasing prevalence of herbivorous guilds, with significantly higher biomass of herbivorous fishes (electronic supplementary material, figure SM2a) and thus potential for cascading effects on reef habitats [4,21,45].

### (b) Shifts in reef biodiversity metrics

Long-term changes were evident for many reef biodiversity metrics investigated: species richness, biomass of fish, abundance of macroinvertebrates, percent cover of algae and CTIs. Strikingly, our results show a clear decline in the richness of macroinvertebrates (−27%) over 27 years, which was most pronounced on sheltered reefs open to fishing. We suggest these declines are caused by warming seas, whether directly via physiological impacts or indirectly through cascading ecological interactions such as increased predation by higher biomass of fishes now present on reefs in all regions of Tasmania. Furthermore, the diminishing abundance and richness on sheltered reefs that were exposed to fishing are also likely driven by fishing pressure.

### (c) Shifts in fish biomass and macroalgal cover

The increase in fish biomass on fished reefs over the duration of our study was associated with changes in both minimum and maximum sea temperatures (figure 4*a,d*). Notwithstanding this, increasing fish biomass may also involve factors other than warming temperature such as increased government regulation of recreational and commercial finfish practices across Tasmania, which also occurred over the study period [46]. Furthermore, monitoring of marine protected areas in Tasmania has demonstrated significant biomass increases within just a few years of protection [47], with further recovery over many decades [48]. In our study, greater warming associated increases in fish biomass occurred on fished reefs than protected reefs, suggesting that overall increases in biomass across target and non-target fish species likely results from a combination of changes in fishing practices and warming seas, with protected reefs showing greater resilience to the effects of warming than fished reefs [26,27,49]. Furthermore, increases in abundance of fish on fished reefs resulted from increases in warm-affinity species, many of them herbivores that are not targeted by fishers. Within marine reserves, increases in warm-affinity species appear resisted by an already well-established fish assemblage including large and abundant predatory species [49]. This further demonstrates that non-climate anthropogenic stressors can interact to pave the way for climate change effects, which can lead to rapid and dramatic reef ecosystem regime-shifts [26].

Canopy macroalgal cover showed a general decrease across Tasmania during the past decade (−20%). However, while a sea urchin population explosion and overgrazing have led to extensive declines in macroalgal cover at depths >10 m in eastern Tasmania in recent decades [45], and herbivorous fishes have increased significantly in the recent decade (see also Vergés *et al*. [4]), the Tasmania-wide decline in macroalgae from approximately 80% to approximately 60% between mid-2000s and 2017–19 was not associated with increase in standing abundances of herbivorous fishes or sea urchins (electronic supplementary material, figure SM7, table SM15a,b). However, biomass of herbivorous fishes was significantly higher across Tasmania and for most of the regions (electronic supplementary material, figure SM2a).

The lower overall cover of macroalgae appears consistent with an interdecadal cycle in canopy cover associated with the Southern Oscillation Index, as previously indicated by Bates *et al*. [14]. Shifts in oceanography, such as that associated with long-term strengthening of the EAC [17], are well known for not only leading to temperature increases but also reductions in nutrients, which can cause reduced macroalgal growth [50,51]. Thus, observed declines in macroalgal cover across Tasmania since 2006 appear more related to an association with the Southern Oscillation Index, rather than increases in herbivores or direct changes in SST. This contrasts with the situation in deeper water (greater than 10 m depth) in eastern Tasmania, which was rarely sampled in our study, but where the warm-affinity *C. rodgersii* has doubled in abundance and the cover of urchin barrens has dramatically increased from 3% to 15% of this coast within 15 years [45]. Given the 10 m depth limit of our study, it is possible that some declining species have undergone thermal retreat to deeper water. This should be investigated using remotely operated sampling methods.

### (d) Shifts in CTI and increasing abundance of warm-affinity species

Examination of changes in relative abundance by species thermal MP indicated that warmer-water suites of fishes and invertebrates are increasing in regions with more pronounced overall trends in CTI. By estimating the changes in relative abundance, we are aware of possible bias if species with low counts disproportionately increased or decreased. However, results did not change noticeably when rarer species (present at between 5% and 10% of sites) were excluded from analysis. Thus, inclusion of rare species added statistical noise to the analysis because of greater stochastic variability among rare species relative to common species, but not the direction or magnitude of the trend. Our analysis by species' relative abundance against their thermal MP revealed an interesting dimension to the changes in the marine communities.

For macroinvertebrates, CTI within zones protected from fishing was negatively related to increasing wave exposure. This pattern could in part be driven by more large lobsters in wave-protected sites within marine reserves, which in turn help to control the greater number of long spine urchins (*C. rodgersii*), which have a high thermal MP. By contrast, fish CTI was positively related to wave exposure, irrespective of level of protection from fishing, which was driven by the presence of a higher abundance of warm-affinity fish species at wave-exposed sites. It is possible that wave-exposed sites had a greater chance of settlement of warm-affinity fish species than more sheltered sites. Wave-sheltered sites tend to be dominated by canopy-forming macroalga, which can buffer the effect of the sun under their canopies during the summer months [28], therefore serving as a refuge to cooler-affinity species and hindering the settlement of warm-affinity species. Differing responses to warming among fishes, invertebrates and macroalgae have also been noted by other authors [28]. We found significant changes in CTI for macroalgae for only the Southeast region, a result that was independent of wave exposure. Cooler-affinity macroalgae were found to persist at wave-exposed sites in a global study of temperate reefs [28].

In a long-term analysis comparing sites inside and outside MPAs across five Tasmanian locations from the early 1990s until 2012, Bates *et al*. [14] found a significant interaction between SST and CTI for all taxonomic groups. The difference between the current and earlier study probably relates to more years included in the time series (13 years of continuous data) and fewer sites. Nonetheless, both studies show the same qualitative increase of CTI for most regions and taxonomic groups.

Warm-affinity fishes, macroinvertebrates and macroalgal species showed greatest increase in relative abundance across all regions. Such increases of warm-affinity species following chronic warming, or following acute heat waves, have been reported in other parts of temperate Australia, with subsequent changes in the trophic structure of the fish communities [11,14,52].

By contrast to warm-affinity species, most macroinvertebrate species preferred cooler waters and generally decreased in abundance, suggesting that warming is at least partially responsible for the rapidly declining richness of macroinvertebrates. While the increased abundance of warmer invertebrates, such as the overgrazing sea urchin *C. rodgersii,* has already had important ecological impacts [21], the potential impacts on the broader functioning of Tasmanian reef ecosystems associated with the decline in cool-affinity species remain unclear. For canopy-forming macroalgae, overall increases in the cover of warmer-affinity species and decreases in cooler-affinity species add further evidence for the ubiquitous impact of warming across all the large and conspicuous components of Tasmanian reef ecosystems.

## 5. Conclusion

Despite apparent stability in reef communities between the 1990s and mid-2000s, recent shifts in reef communities indicate that ongoing and chronic coastal warming is affecting the abundance of species on Tasmanian temperate reefs. The greatest multi-decadal changes were evident for fish communities, followed by macroinvertebrates, but were slight for macroalgae. The observed patterns in the marine communities could in part be driven by differences in dispersal and settlement, as well as the mix of broader or narrower thermal range affinity species within each taxonomic group. Changes have not been uniform across taxonomic groups, regions, levels of wave exposure or protection from fishing. Ongoing warming will presumably continue to drive cooler-affinity species out of temperate reef communities and promote dominance of warmer-affinity species, with associated changes in functional composition. Furthermore, pronounced shifts in macroalgal communities are expected, including potentially complete local loss of kelp beds due to overgrazing by warmer-affinity species in some regions. While warming-driven reorganization is occurring, the long-term dampening effect we observed from protection in marine reserves provides a reminder that climate and local anthropogenic stressors do not act independently, and that local management action can improve resilience of temperate reef ecosystems into the warming future.

## Data Availability

Data used for this paper can be accessed at: https://portal.aodn.org.au/search. Type ATRC in the search box in the bottom left of this portal. Tables and figures derived from this data are provided in electronic supplementary material [53].
